# Supplementary material for: Induced-fit expansion and contraction of a self-assembled nanocube finely responding to neutral and anionic guests
Source: Nat Commun. 2018 Oct 31;9:4530. doi: 10.1038/s41467-018-06874-y (PMC6208372; doi:10.1038/s41467-018-06874-y)
Supplement: Supplementary file 1 — Supplementary Information [file 41467_2018_6874_MOESM1_ESM.pdf]

Supplementary Figures

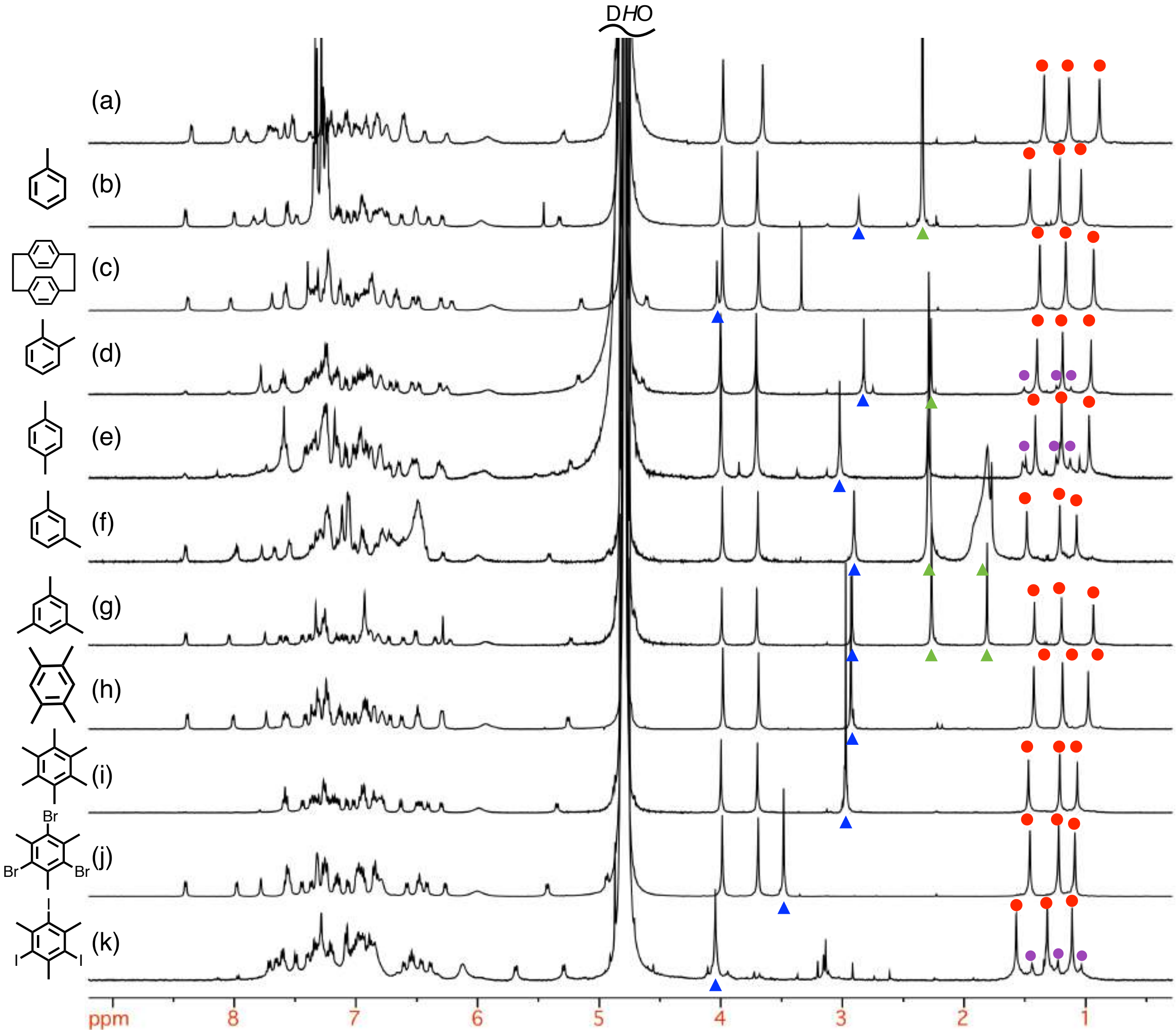

Supplementary Figure 1. ^{1}H NMR spectra of the host-guest complexes between $\mathbf{1}_6$ and aromatic guest molecules (500 MHz, 298 K, D_2O, $[\mathbf{1}]_{total}$ = 1.0 mM). (a) $\mathbf{1}_6$. (b) toluene$_3$@$\mathbf{1}_6$. (c) PC@$\mathbf{1}_6$, PC indicates [2.2]paracyclophane. (d) *o*-xylene$_2$@$\mathbf{1}_6$, a trace amount of *o*-xylene$_3$@$\mathbf{1}_6$ is indicated by violet solid circles. (e) *p*-xylene$_2$@$\mathbf{1}_6$, a trace amount of *p*-xylene$_3$@$\mathbf{1}_6$ is indicated by violet solid circles. (f) *m*-xylene$_3$@$\mathbf{1}_6$. (g) mesitylene$_2$@$\mathbf{1}_6$. (h) durene$_2$@$\mathbf{1}_6$. (i) hexamethylbenzene$_2$@$\mathbf{1}_6$. (j) TBM$_2$@$\mathbf{1}_6$, TBM indicates 1,3,5-tribromomesitylene. (k) 1,3,5-triiodomesitylene$_2$@$\mathbf{1}_6$, a trace amount of 1,3,5-triiodomesitylene@$\mathbf{1}_6$ is indicated by violet solid circles. Red solid circles indicate the methyl signals of the *p*-tolyl methyl groups in $\mathbf{1}_6$. Blue and green solid triangles indicate the signals of encapsulated and free guests, respectively. Two signals were observed for liquid free guest, mesitylene and *m*-xylene, that derived from the water-soluble molecules and the undissolved bulky molecules.

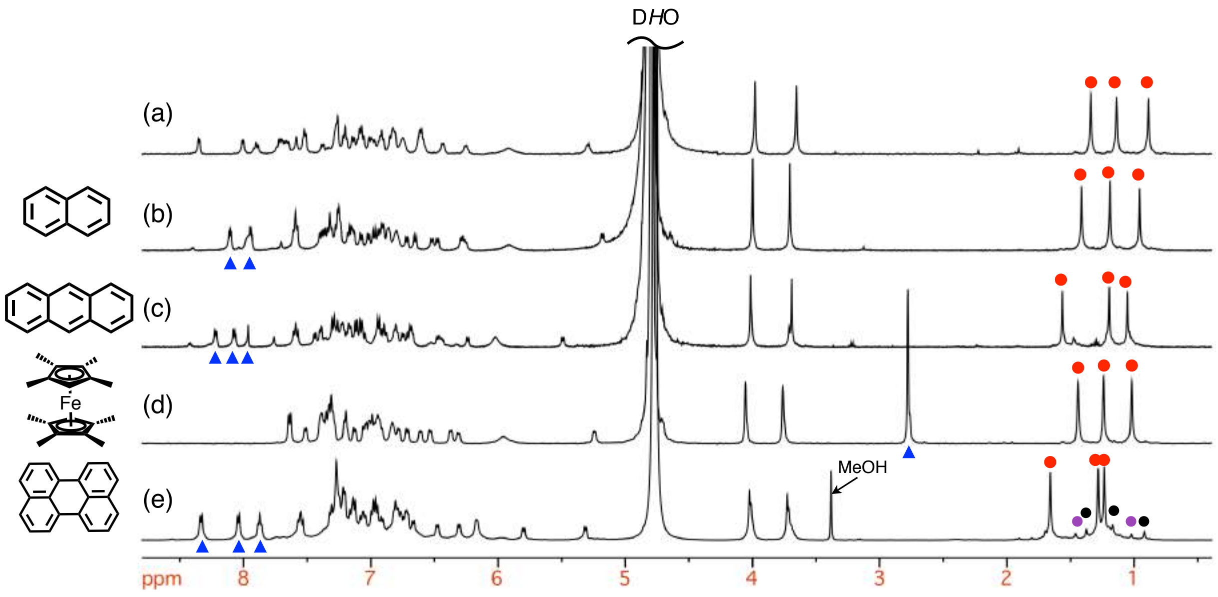

Supplementary Figure 2. ^{1}H NMR spectra of the host-guest complexes between $\mathbf{1}_6$ and large aromatic molecules (500 MHz, 298 K, D_2O, $[\mathbf{1}]_{total}$ = 1.0 mM). (a) $\mathbf{1}_6$. (b) naphthalene$_2$@$\mathbf{1}_6$. (c) anthracene$_2$@$\mathbf{1}_6$. (d) DF@$\mathbf{1}_6$, DF indicates decamethylferrocene. (e) perylene$_2$@$\mathbf{1}_6$, a trace amount of $\mathbf{1}_6$ and perylene@$\mathbf{1}_6$ are indicated by black and violet solid circles, respectively. Red solid circles indicate the *p*-tolyl methyl groups of $\mathbf{1}_6$. Blue solid triangles indicate the signals of encapsulated guests.

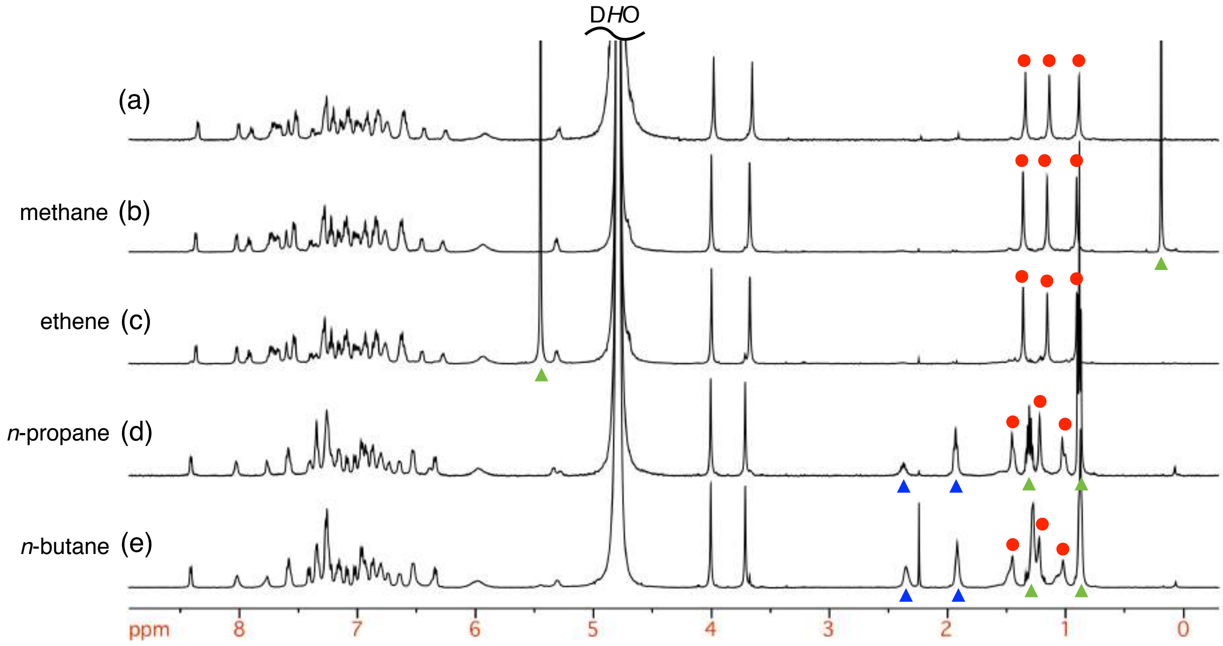

Supplementary Figure 3. ^{1}H NMR spectra of the host-guest complexes between $\mathbf{1}_6$ and C1 – C4 hydrocarbons (500 MHz, 298 K, D_2O, $[\mathbf{1}]_{total}$ = 1.0 mM). (a) $\mathbf{1}_6$. (b) $\mathbf{1}_6$ in the presence of methane. (c) $\mathbf{1}_6$ in the presence of ethene. (d) *n*-propane$_3$@$\mathbf{1}_6$. (e) *n*-butane$_3$@$\mathbf{1}_6$. Red solid circles indicate the *p*-tolyl methyl groups of $\mathbf{1}_6$. Blue and green solid triangles indicate the signals of encapsulated and free guests, respectively.

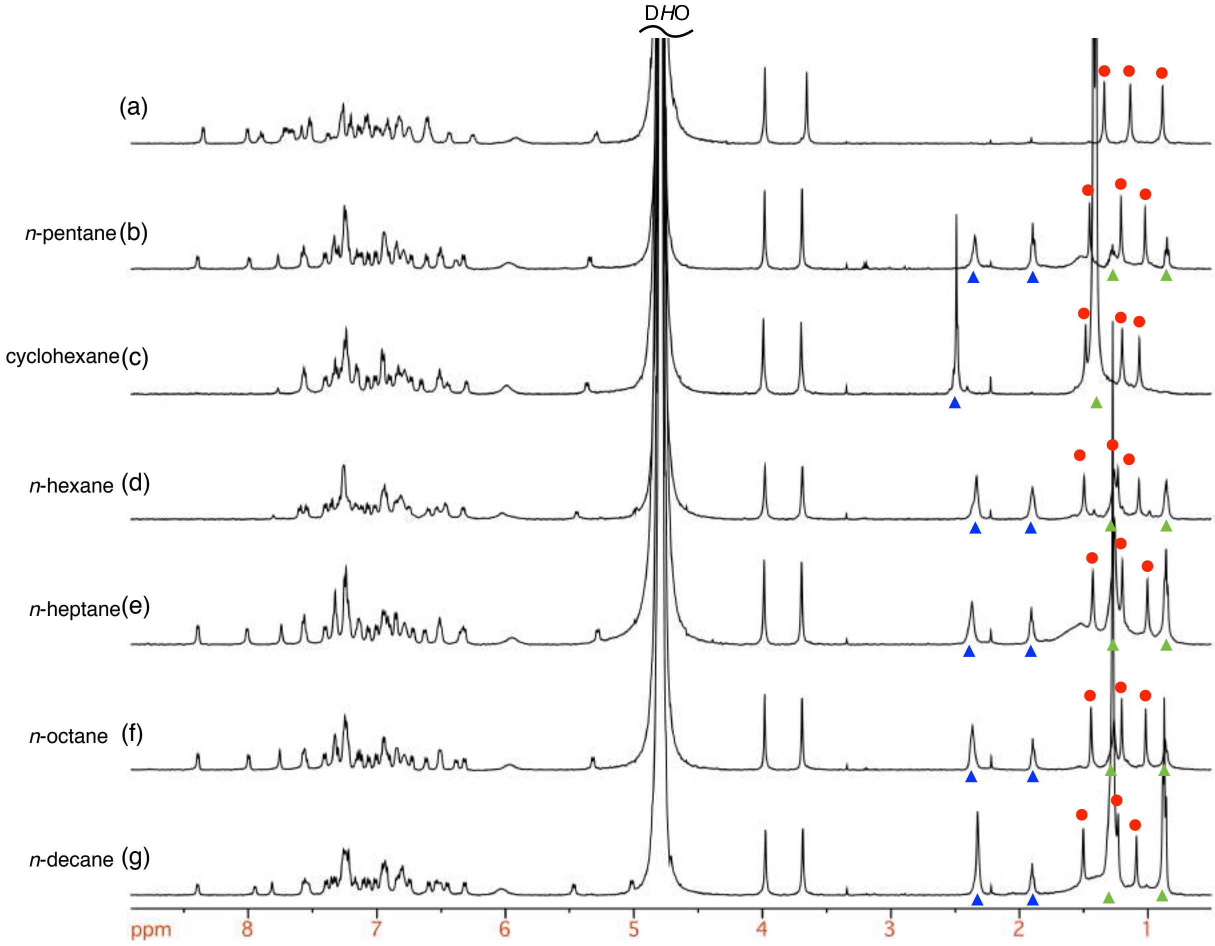

Supplementary Figure 4. ^{1}H NMR spectra of the host-guest complexes between $\mathbf{1}_6$ and C5 – C10 alkanes (500 MHz, 298 K, D_2O, $[\mathbf{1}]_{total}$ = 1.0 mM). (a) $\mathbf{1}_6$. (b) *n*-pentane$_3$@$\mathbf{1}_6$. (c) cyclohexane$_3$@$\mathbf{1}_6$. (d) *n*-hexane$_3$@$\mathbf{1}_6$. (e) *n*-heptane$_2$@$\mathbf{1}_6$. (f) *n*-octane$_2$@$\mathbf{1}_6$. (g) *n*-decane$_2$@$\mathbf{1}_6$. Red solid circles indicate the *p*-tolyl methyl groups of $\mathbf{1}_6$. Blue and green solid triangles indicate the signals of encapsulated and free guests, respectively.

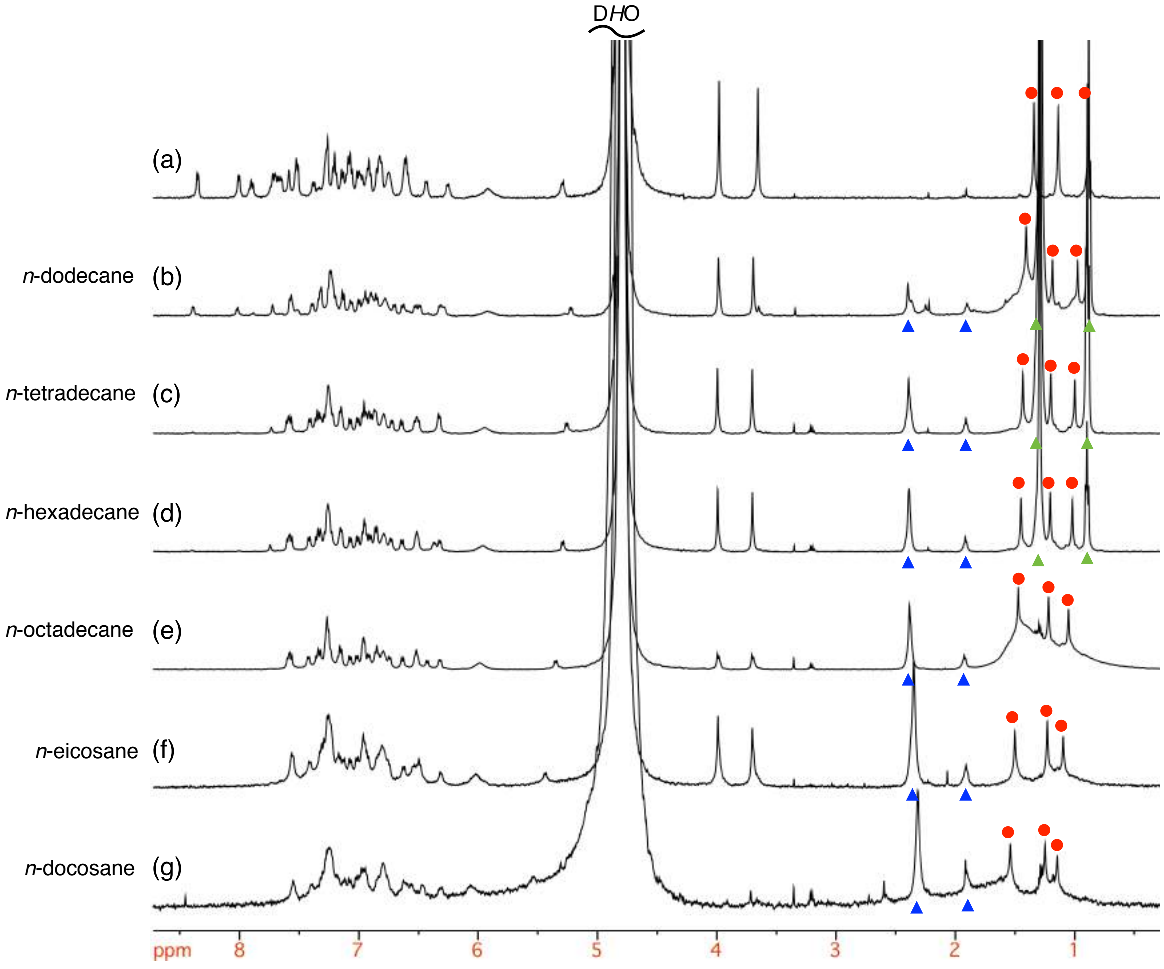

Supplementary Figure 5. ^{1}H NMR spectra of the host-guest complexes between $\mathbf{1}_6$ and C12 – C22 alkanes (500 MHz, 298 K, D_2O, $[\mathbf{1}]_{total}$ = 1.0 mM). (a) $\mathbf{1}_6$. (b) *n*-dodecane@$\mathbf{1}_6$. (c) *n*-tetradecane@$\mathbf{1}_6$. (d) *n*-hexadecane@$\mathbf{1}_6$. (e) *n*-octadecane@$\mathbf{1}_6$. (f) *n*-eicosane@$\mathbf{1}_6$. (g) *n*-docosane@$\mathbf{1}_6$. Red solid circles indicate the *p*-tolyl methyl groups of $\mathbf{1}_6$. Blue and green solid triangles indicate the signals of encapsulated and free guests, respectively.

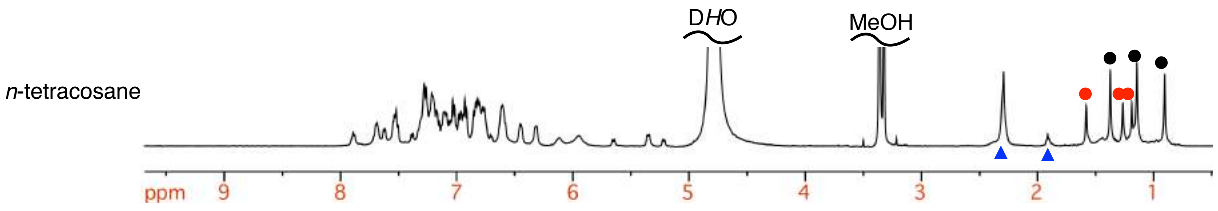

Supplementary Figure 6. ^{1}H NMR spectra of the host-guest complexation between $\mathbf{1}_6$ and *n*-tetracosane (500 MHz, 298 K, D_2O, $[\mathbf{1}]_{total}$ = 1.0 mM). Red and black solid circles indicate the signals of the *p*-tolyl methyl groups of *n*-tetracosane@$\mathbf{1}_6$ and $\mathbf{1}_6$, respectively. Blue solid triangles indicate the signals of the encapsulated *n*-tetracosane.

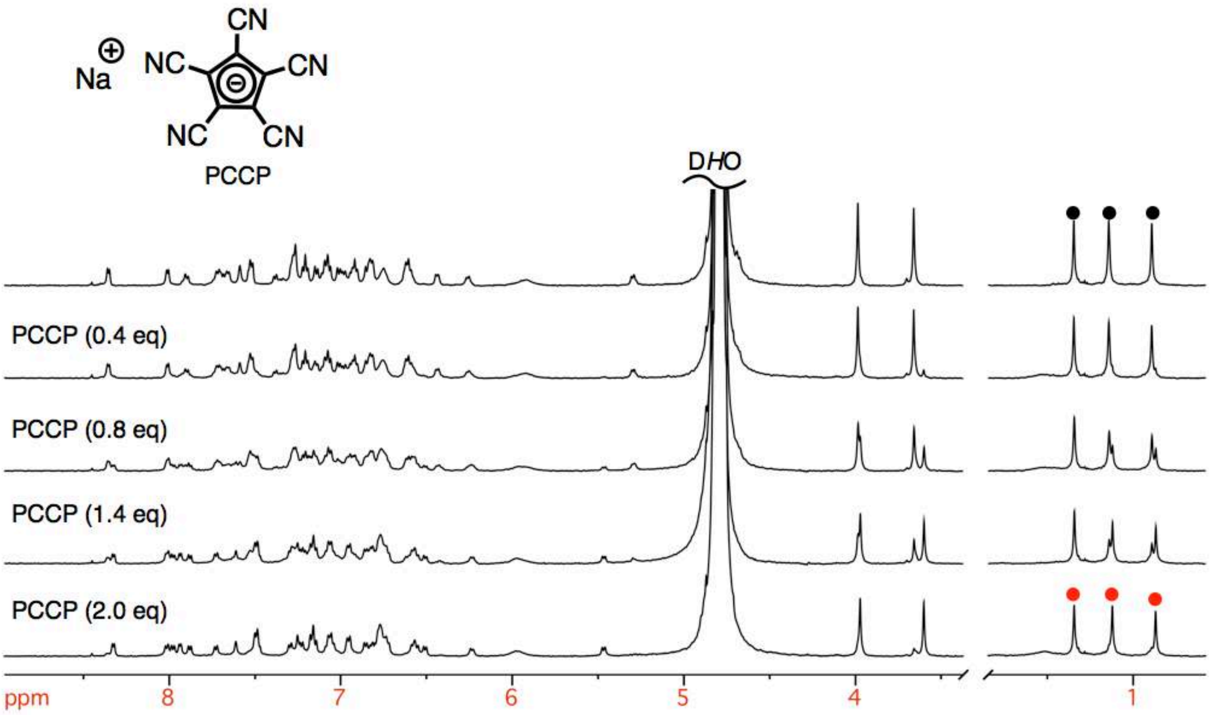

Supplementary Figure 7. ^{1}H NMR titration experiments of PCCP against $\mathbf{1}_6$ (500 MHz, 298 K, D_2O, $[\mathbf{1}]_{total}$ = 1.0 mM, PCCP indicates pentacyanocyclopentadienide). Red and black solid circles indicate the signals of the *p*-tolyl methyl signals of $PCCP_2@\mathbf{1}_6$ and $\mathbf{1}_6$, respectively.

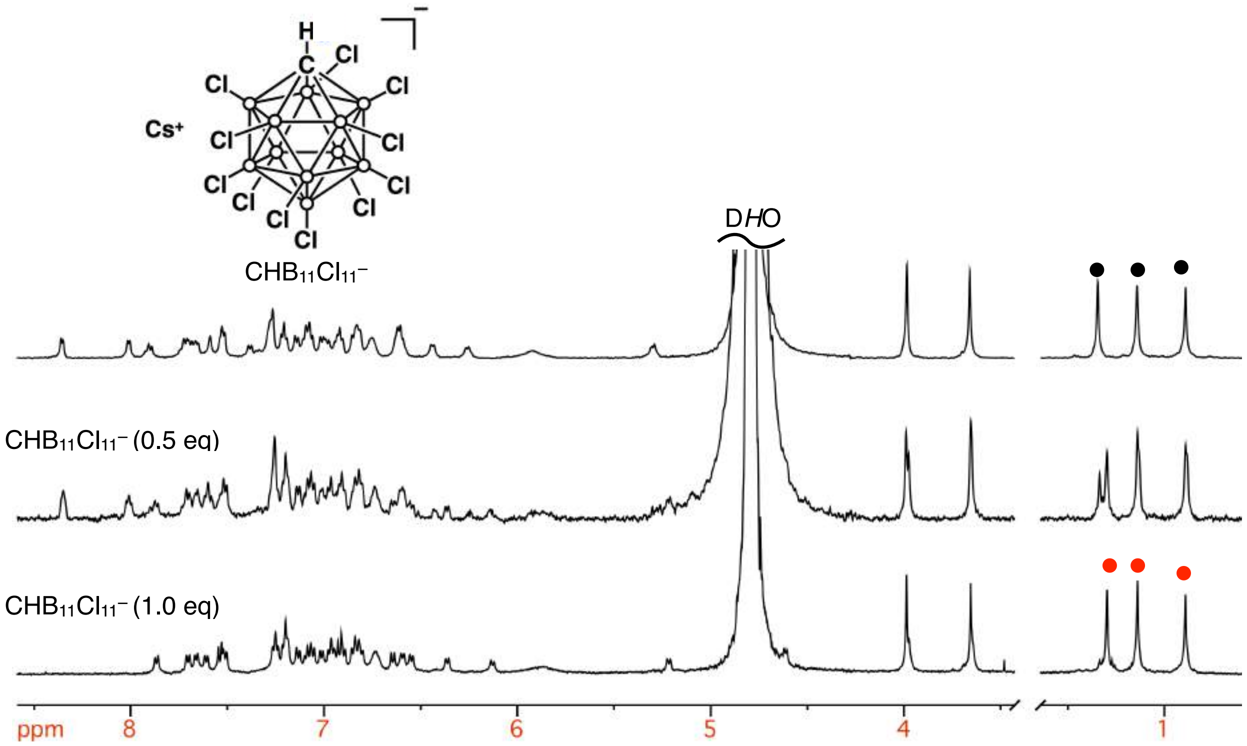

Supplementary Figure 8. ^{1}H NMR titration experiments of $CHB_{11}Cl_{11}^{-}$ (CB) against $\mathbf{1}_6$ (500 MHz, 298 K, D_2O, $[\mathbf{1}]_{total}$ = 1.0 mM). Red and black solid circles indicate the *p*-tolyl methyl signals of $CHB_{11}Cl_{11}^{-}@\mathbf{1}_6$ and $\mathbf{1}_6$, respectively.

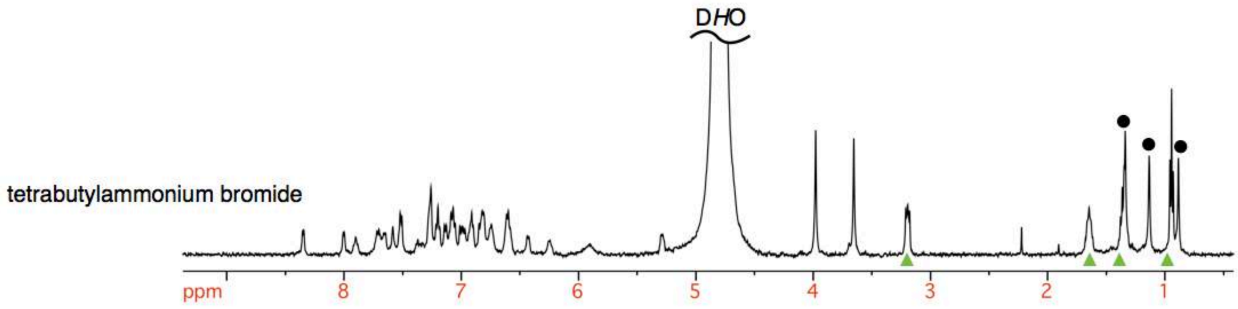

Supplementary Figure 9. ^{1}H NMR spectra of the mixture of $\mathbf{1}_6$ and tetra-*n*-butylammonium (TBA) bromide (500 MHz, 298 K, D_2O, $[\mathbf{1}]_{total}$ = 1.0 mM). Black and green solid circles indicate the signals of the *p*-tolyl methyl groups of free $\mathbf{1}_6$ and TBA, respectively.

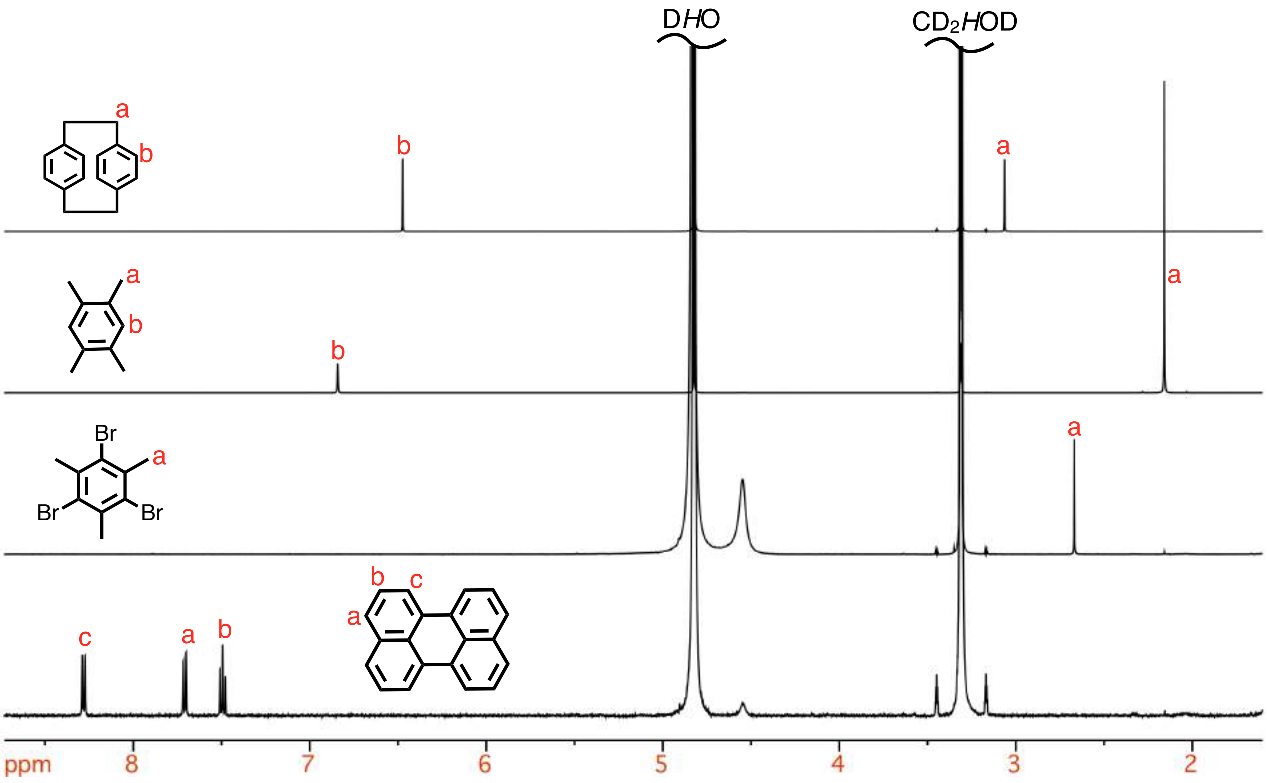

Supplementary Figure 10. ^{1}H NMR spectra of selected guest molecules (500 MHz, 298 K, CD_3OD).

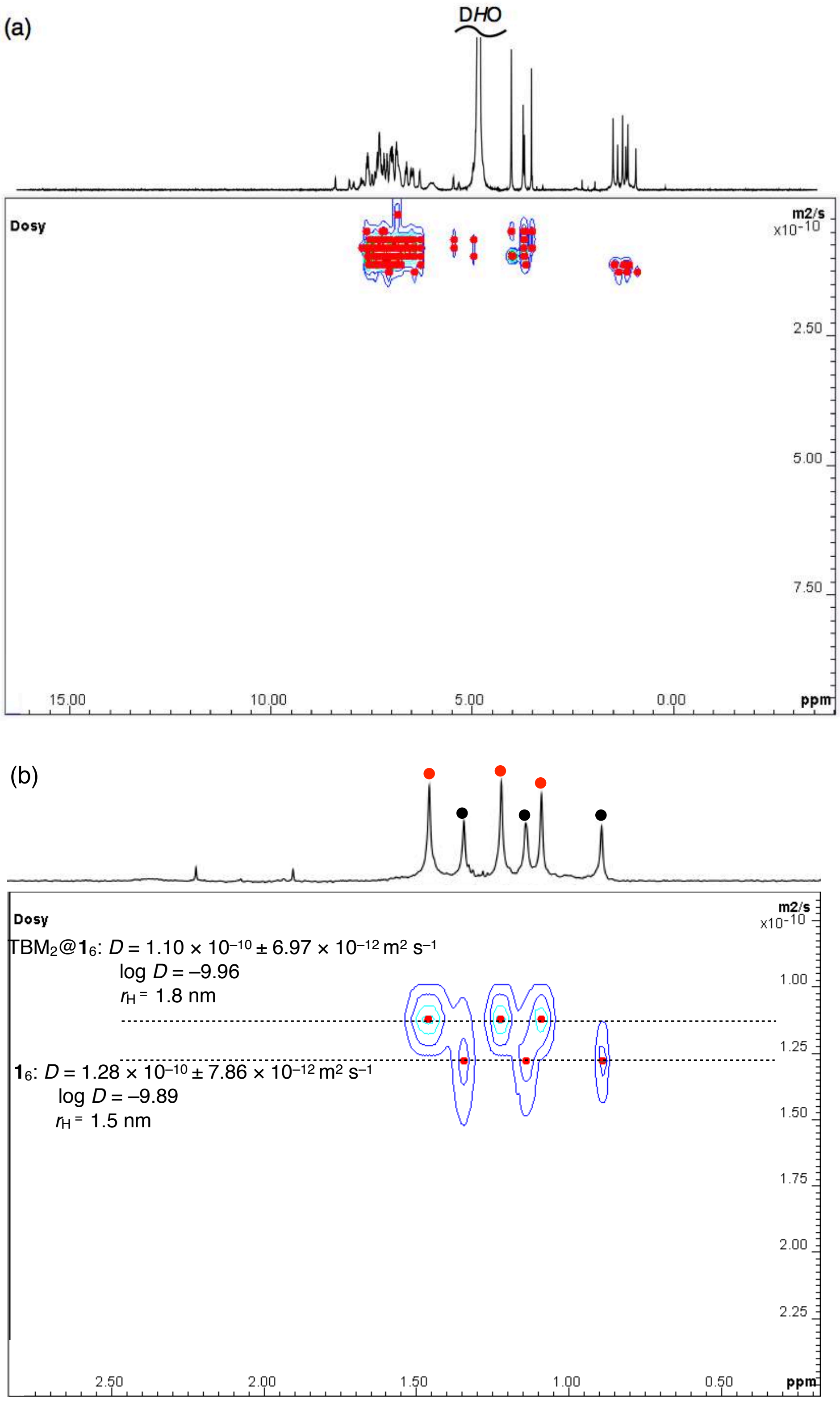

(a)
DHO
Dosy
m2/s
x10-10
2.50
5.00
7.50
15.00
10.00
5.00
0.00
ppm
(b)
Dosy
m2/s
x10-10
TBM$_2$@**1**$_6$: D = 1.10 × 10^{-10} ± 6.97 × 10^{-12} m^2 s^{-1}
log D = −9.96
r_H = 1.8 nm
1$_6$: D = 1.28 × 10^{-10} ± 7.86 × 10^{-12} m^2 s^{-1}
log D = −9.89
r_H = 1.5 nm
1.00
1.25
1.50
1.75
2.00
2.25
2.50
2.00
1.50
1.00
0.50
ppm

(c)

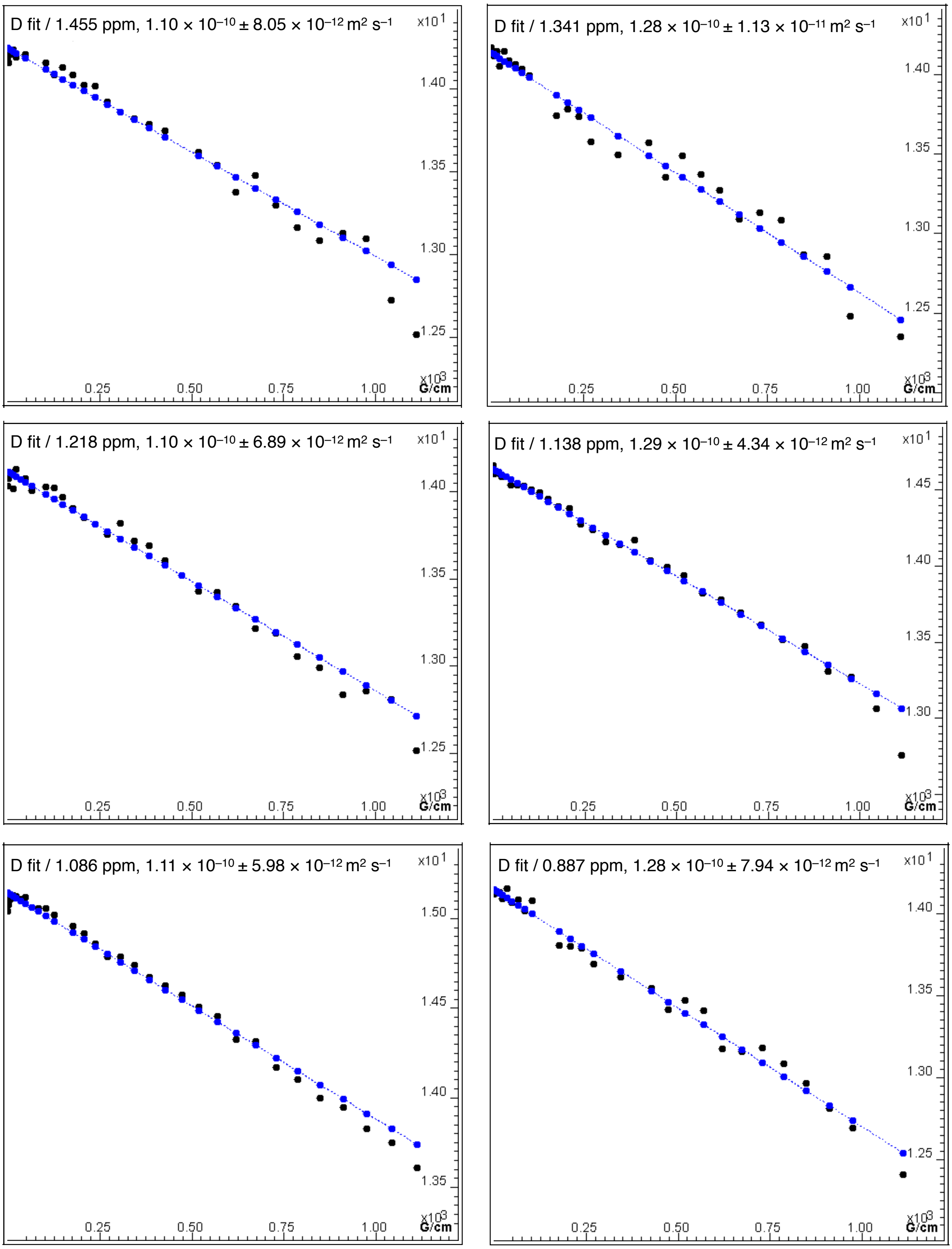

Supplementary Figure 11. ^{1}H DOSY spectra of a mixture of the $\mathbf{1}_6$ and TBM$_2$@$\mathbf{1}_6$ (500 MHz, 298 K, D_2O, [**1**]$_{total}$ = 1.0 mM). (a) A full spectrum. (b) Aliphatic region from 0 to 3 ppm. TBM indicates 1,3,5-tribromomesitylene. Red and black solid circles indicate the *p*-tolyl methyl signals of TBM$_2$@$\mathbf{1}_6$ and $\mathbf{1}_6$, respectively. The diffusion coefficient of TBM$_2$@$\mathbf{1}_6$ and $\mathbf{1}_6$ was determined by the average *D* value of the three *p*-tolyl methyl groups of TBM$_2$@$\mathbf{1}_6$ and $\mathbf{1}_6$, respectively. r_H indicates hydrodynamic radius, which was determined by using the Stokes-Einstein equation, $D = kT/6\pi\eta r_H$, where k is the Boltzmann constant, T is the temperature, and η is the viscosity of the solution. (c) The fitting curves of the *p*-tolyl methyl groups of TBM$_2$@$\mathbf{1}_6$ and $\mathbf{1}_6$.

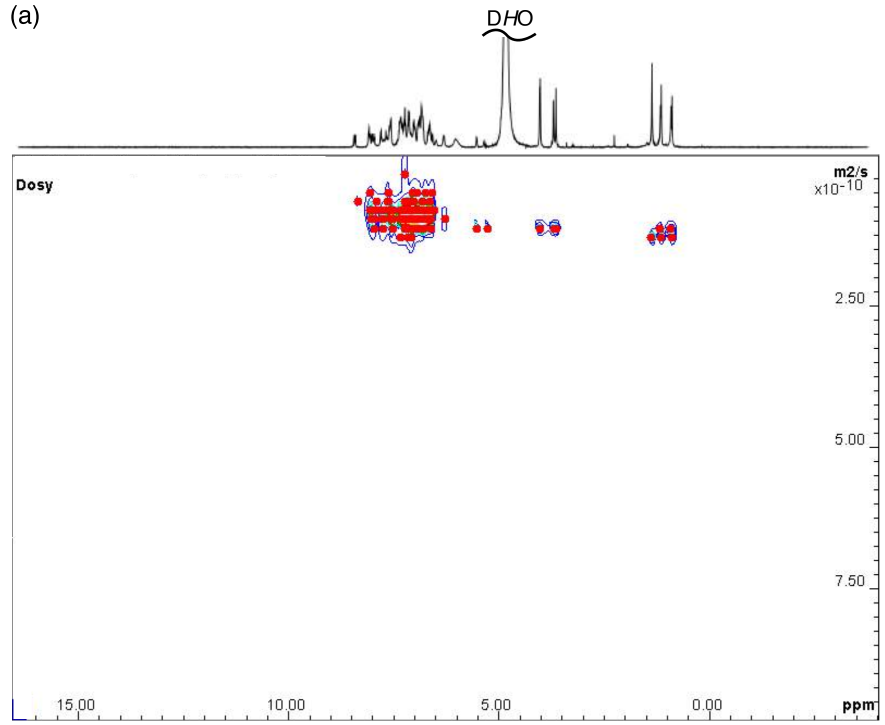

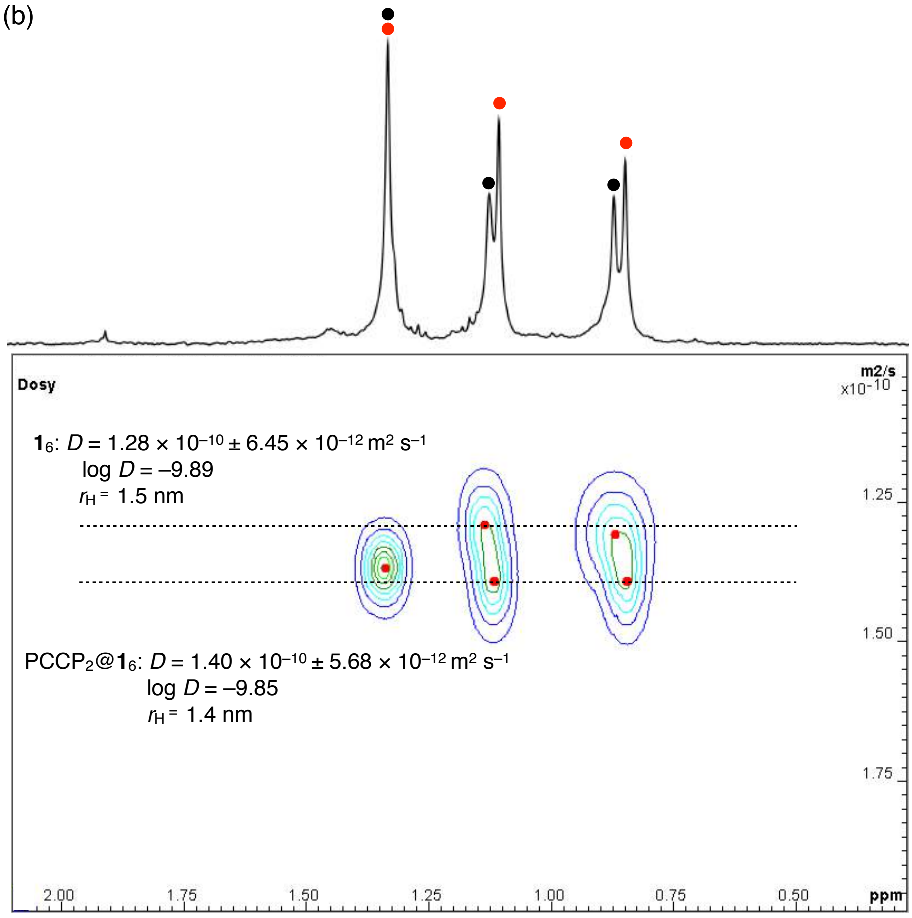

1$_6$: $D = 1.28 \times 10^{-10} \pm 6.45 \times 10^{-12}$ m^2 s^{-1}
log $D = -9.89$
r_H = 1.5 nm

PCCP$_2$@**1**$_6$: $D = 1.40 \times 10^{-10} \pm 5.68 \times 10^{-12}$ m^2 s^{-1}
log $D = -9.85$
r_H = 1.4 nm

(c)

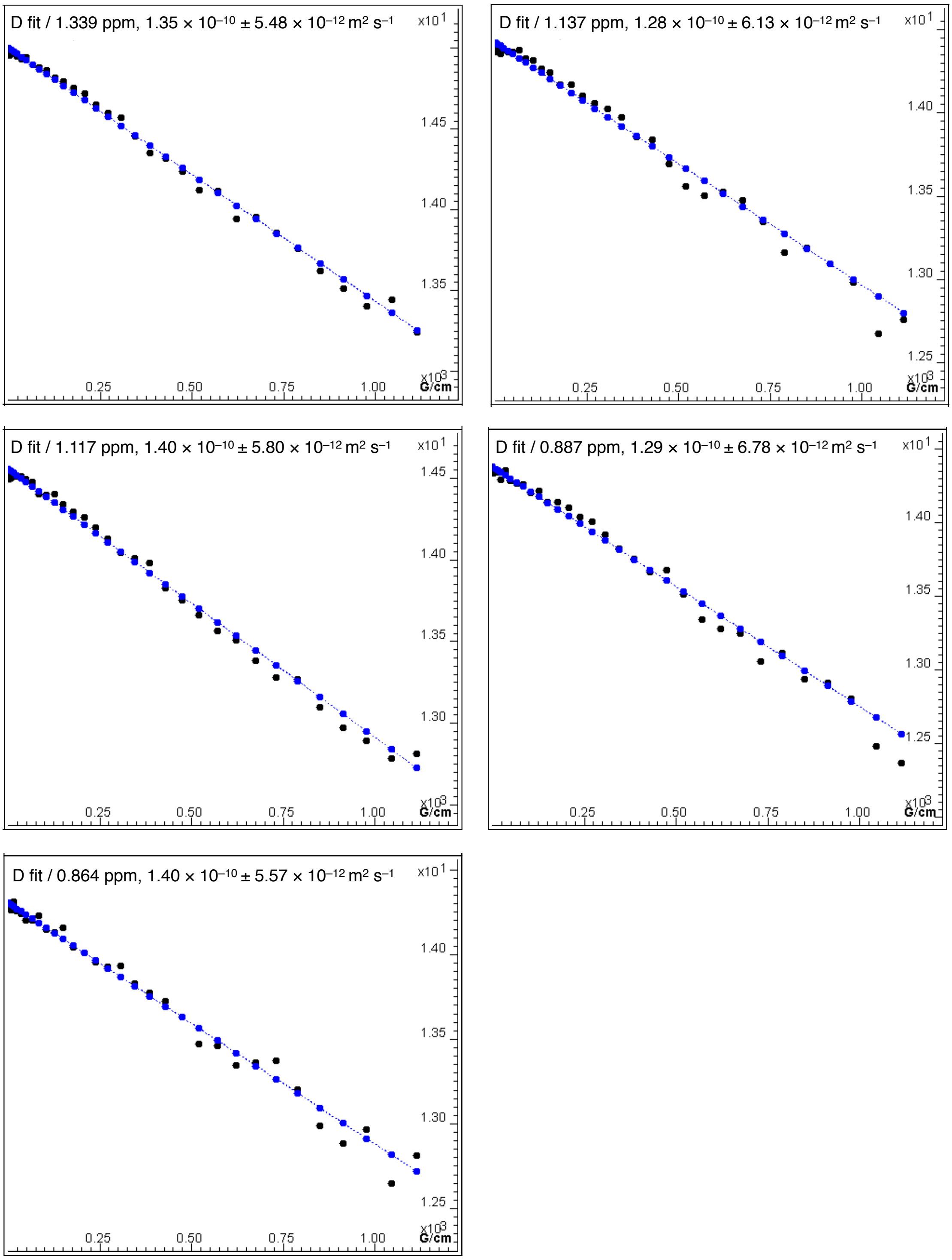

Supplementary Figure 12. ^{1}H DOSY spectra of a mixture of $\mathbf{1}_6$ and PCCP$_2$@$\mathbf{1}_6$ nanocubes (500 MHz, 298 K, D_2O, [**1**]$_{total}$ = 1.0 mM). (a) A full spectrum. (b) Aliphatic region from –1 to 3 ppm. PCCP indicates pentacyanocyclopentadienide. Red and black solid circles indicate the *p*-tolyl methyl signals of PCCP$_2$@$\mathbf{1}_6$ and $\mathbf{1}_6$, respectively. The diffusion coefficient of PCCP$_2$@$\mathbf{1}_6$ and $\mathbf{1}_6$ was determined by the average *D* value of the two upfield signals of the *p*-tolyl methyl groups of PCCP$_2$@$\mathbf{1}_6$ (1.117 ppm, 0.864 ppm) and $\mathbf{1}_6$ (1.137 ppm, 0.887 ppm), respectively. The ^{1}H NMR signal at 1.339 ppm is composed of 65% PCCP$_2$@$\mathbf{1}_6$ and 35% $\mathbf{1}_6$ based on the integral value the *p*-tolyl methyl groups and its diffusion coefficient is 1.35 × 10^{-10} m^2s^{-1}, which is between the diffusion coefficient of PCCP$_2$@$\mathbf{1}_6$ and $\mathbf{1}_6$. r_H indicates hydrodynamic radius, which was determined by using the Stokes-Einstein equation. (c) The fitting curves of the *p*-tolyl methyl groups of TBM$_2$@$\mathbf{1}_6$ and $\mathbf{1}_6$.

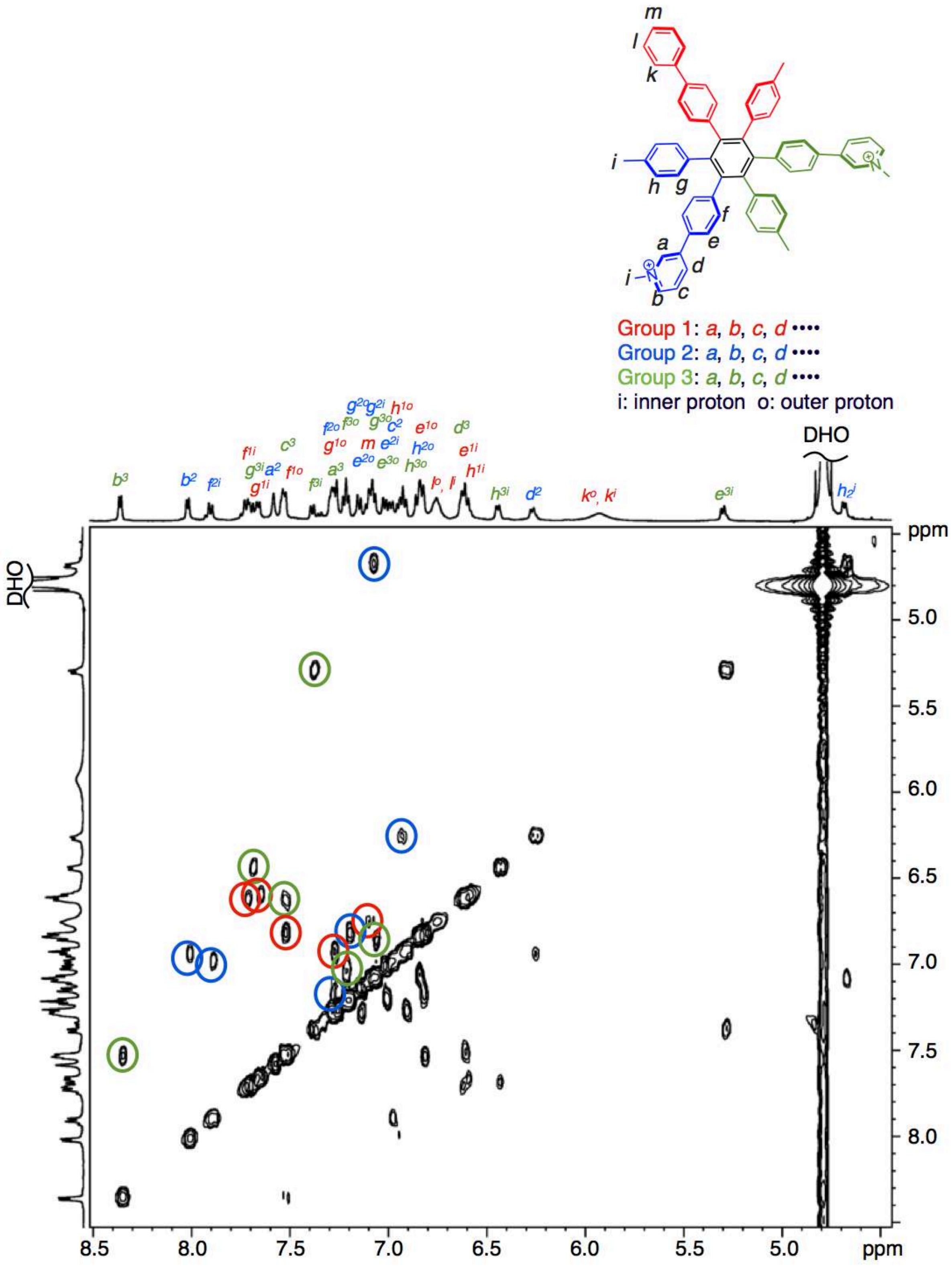

Supplementary Figure 13. ^{1}H–^{1}H COSY spectra of $\mathbf{1}_6$ nanocube (500 MHz, D_2O, 298 K). Red, blue, and green circles indicate *ortho* coupling between protons, which belong to groups *1, 2*, and *3*, respectively.

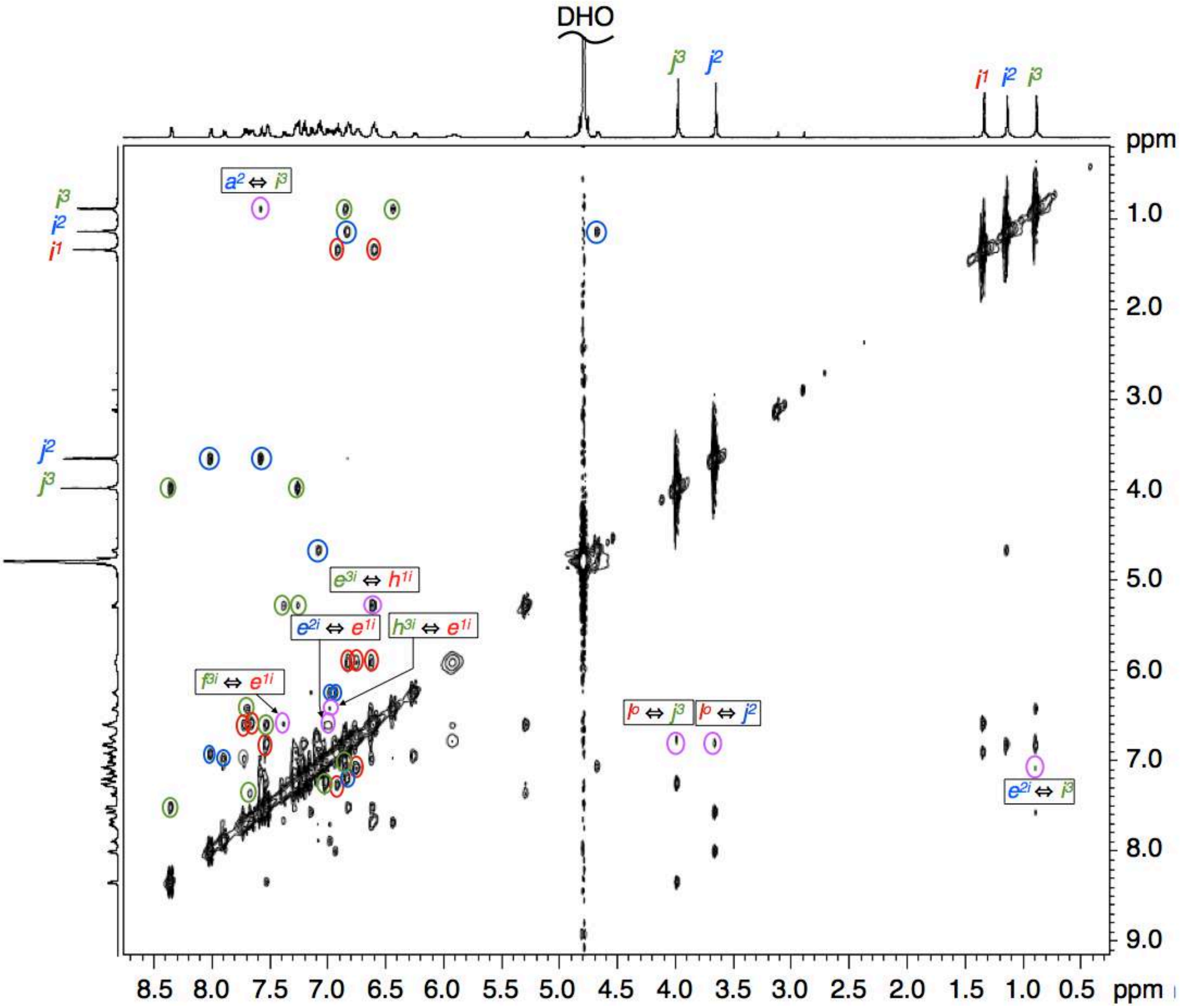

Supplementary Figure 14. ^{1}H–^{1}H NOESY spectrum of the $\mathbf{1}_6$ nanocube (500 MHz, D_2O, 298 K, mixing time = 0.5 s). Magenta circles indicate intermolecular NOE. Red, blue, and green circles indicate intramolecular NOEs between protons belonging to groups *1, 2*, and *3*, respectively.

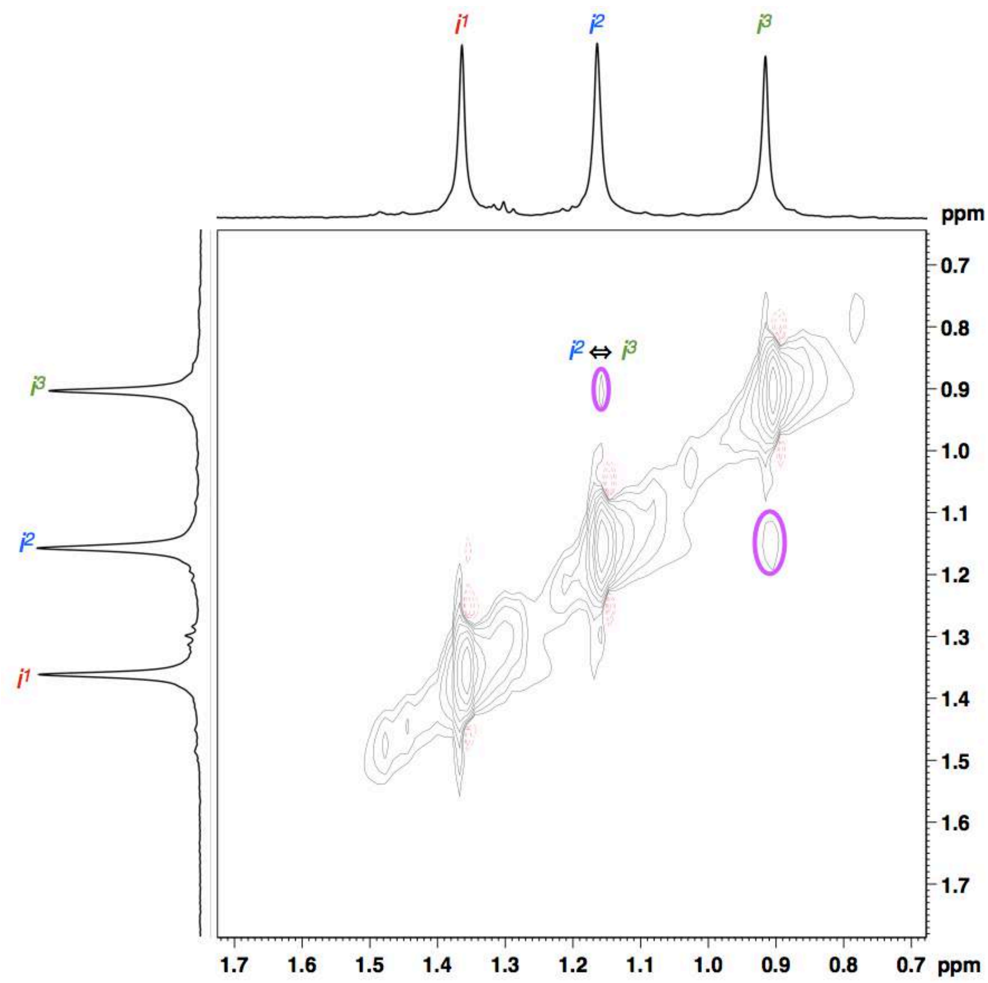

Supplementary Figure 15. ^1H–^1H NOESY spectrum of the $\mathbf{1}_6$ nanocube (500 MHz, D_2O, 298 K, mixing time = 0.2 s). Magenta circles indicate intermolecular NOE between i^2 and i^3.

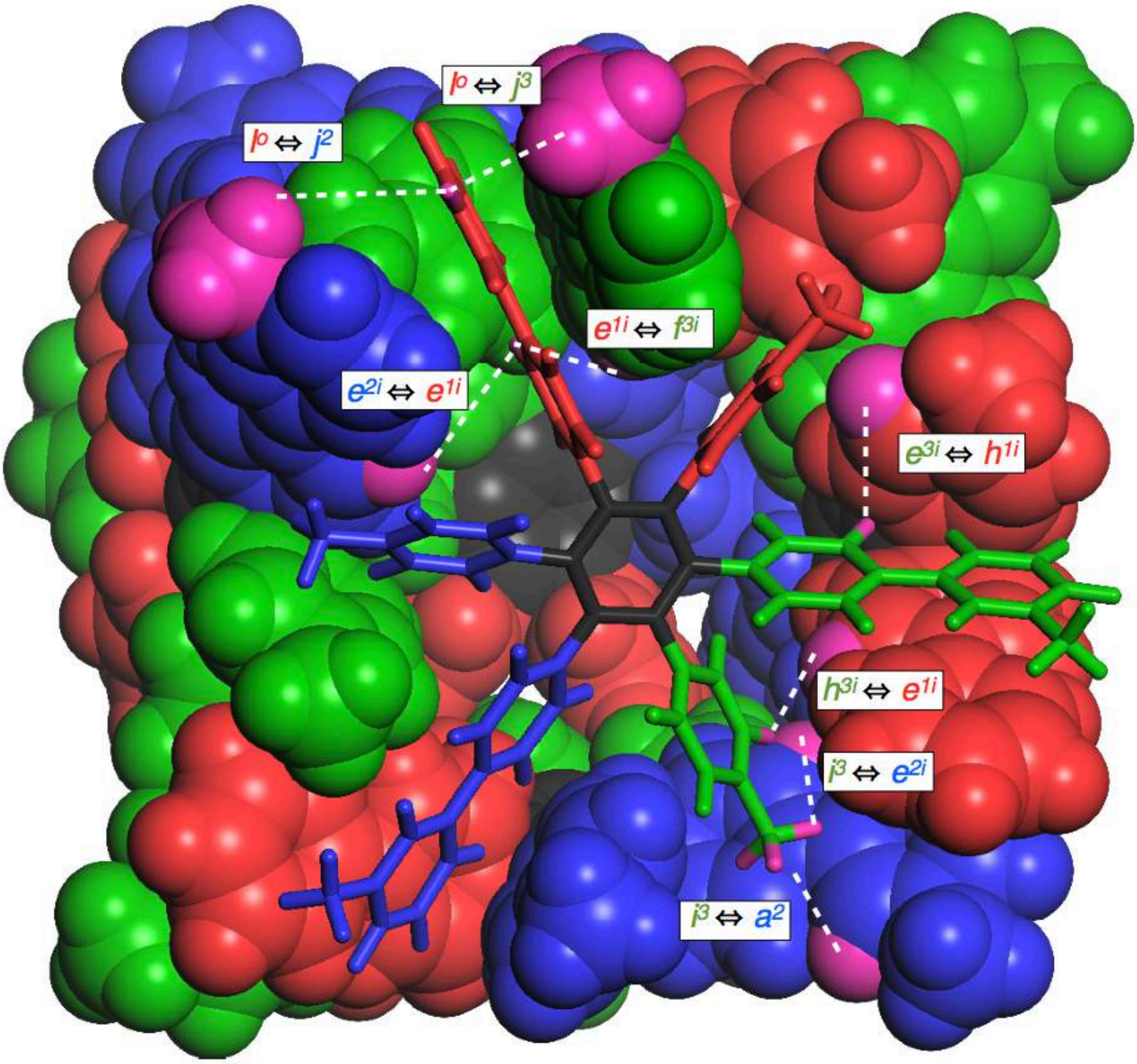

Supplementary Figure 16. The structure of $\mathbf{1}_6$ indicative of the intermolecular proton pairs where NOE cross peaks were observed (See Supplementary Figure 14). White dotted lines denote the pairs of protons (colored in magenta) where intermolecular NOE cross-peaks were observed.

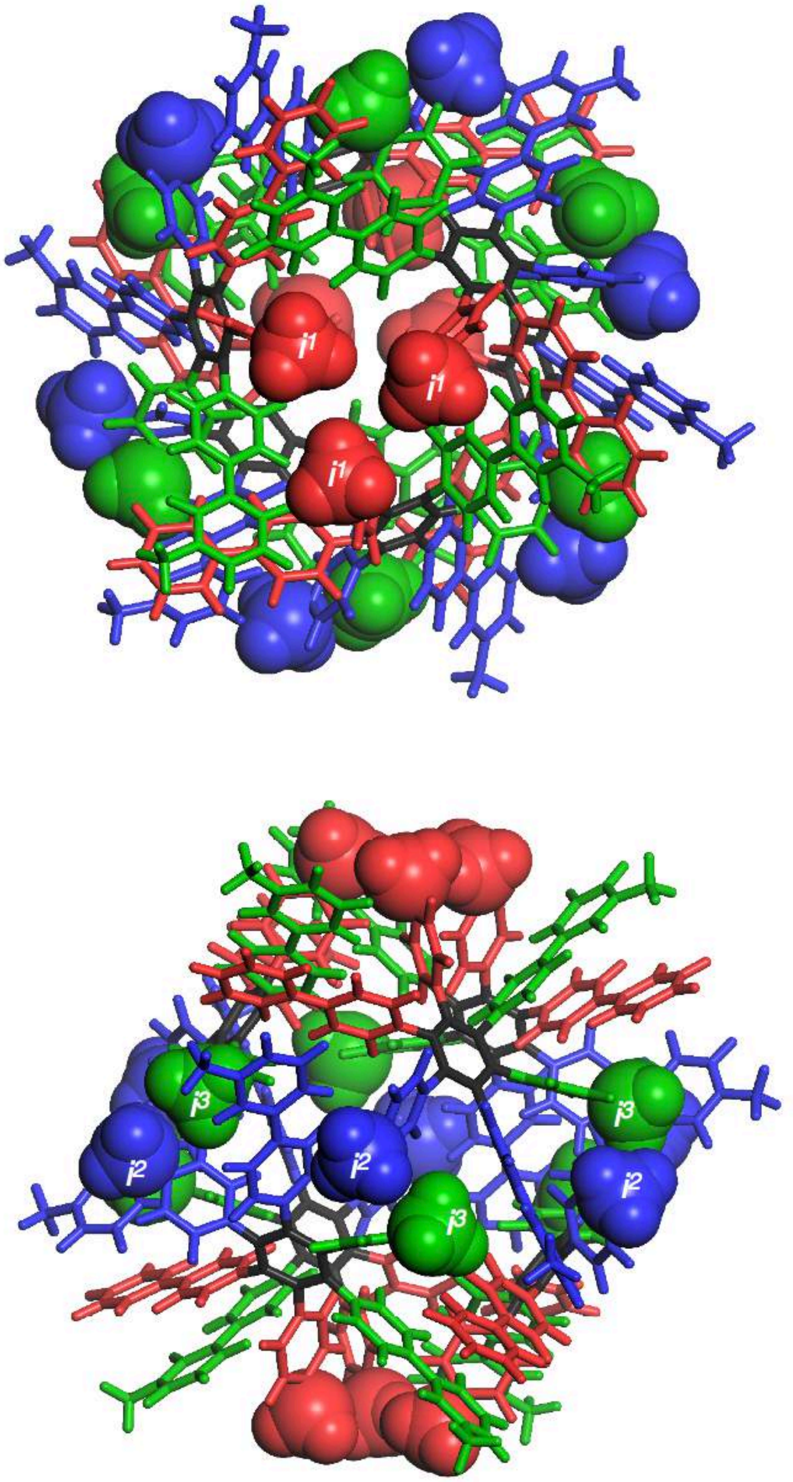

Supplementary Figure 17. Schematic description of the positions of three *p*-tolyl methyl groups, which are shown as space-filling model. Red methyl groups (i^1) are close to each other, while Blue methyl groups (i^2) and green ones (i^3) are close to each other.

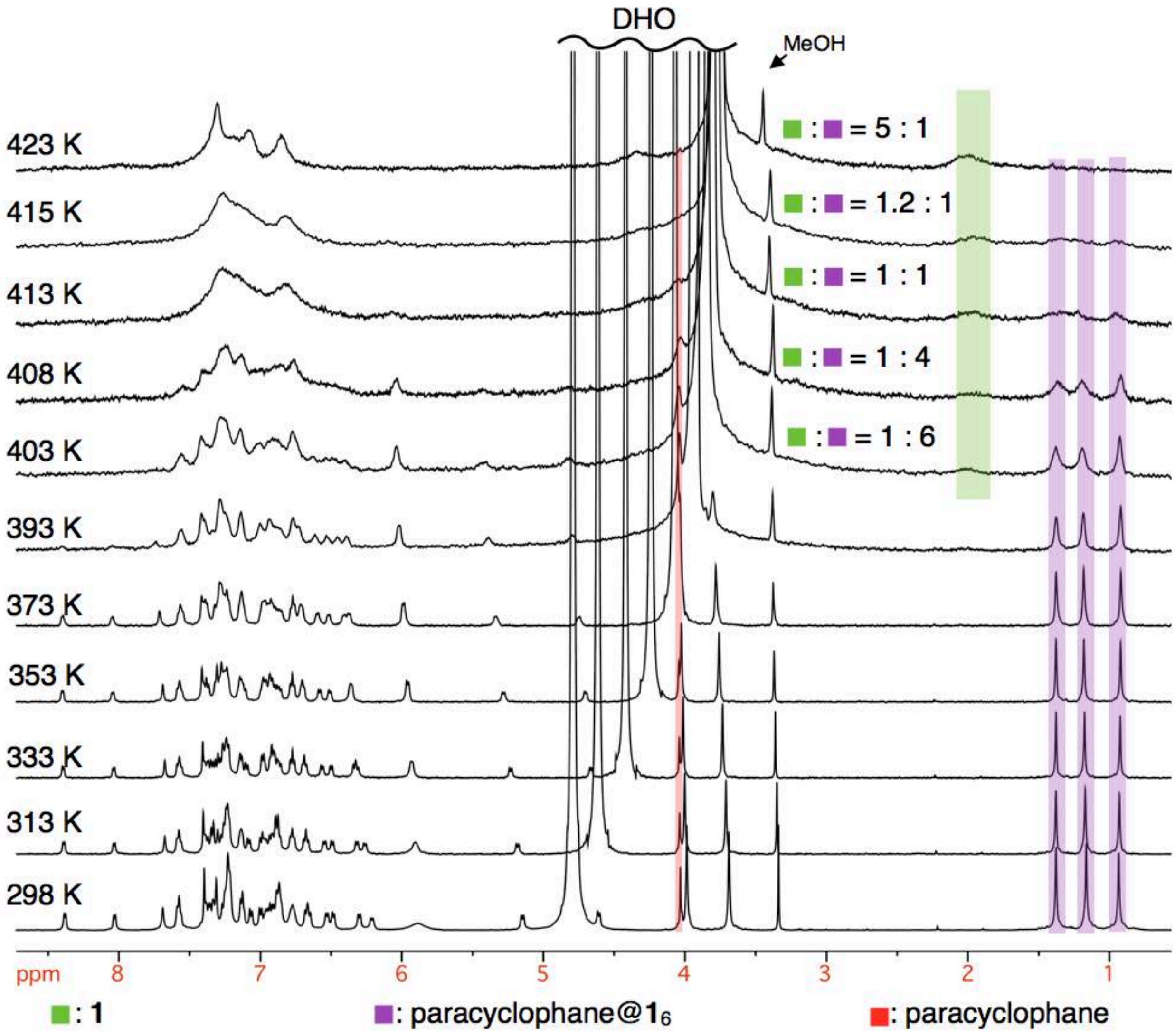

Supplementary Figure 18. Variable temperature ^{1}H NMR spectra of PC@$\mathbf{1}_6$ (500 MHz, D_2O, $[\mathbf{1}]_{total}$ = 1.0 mM). PC indicates [2.2]paracyclophane.

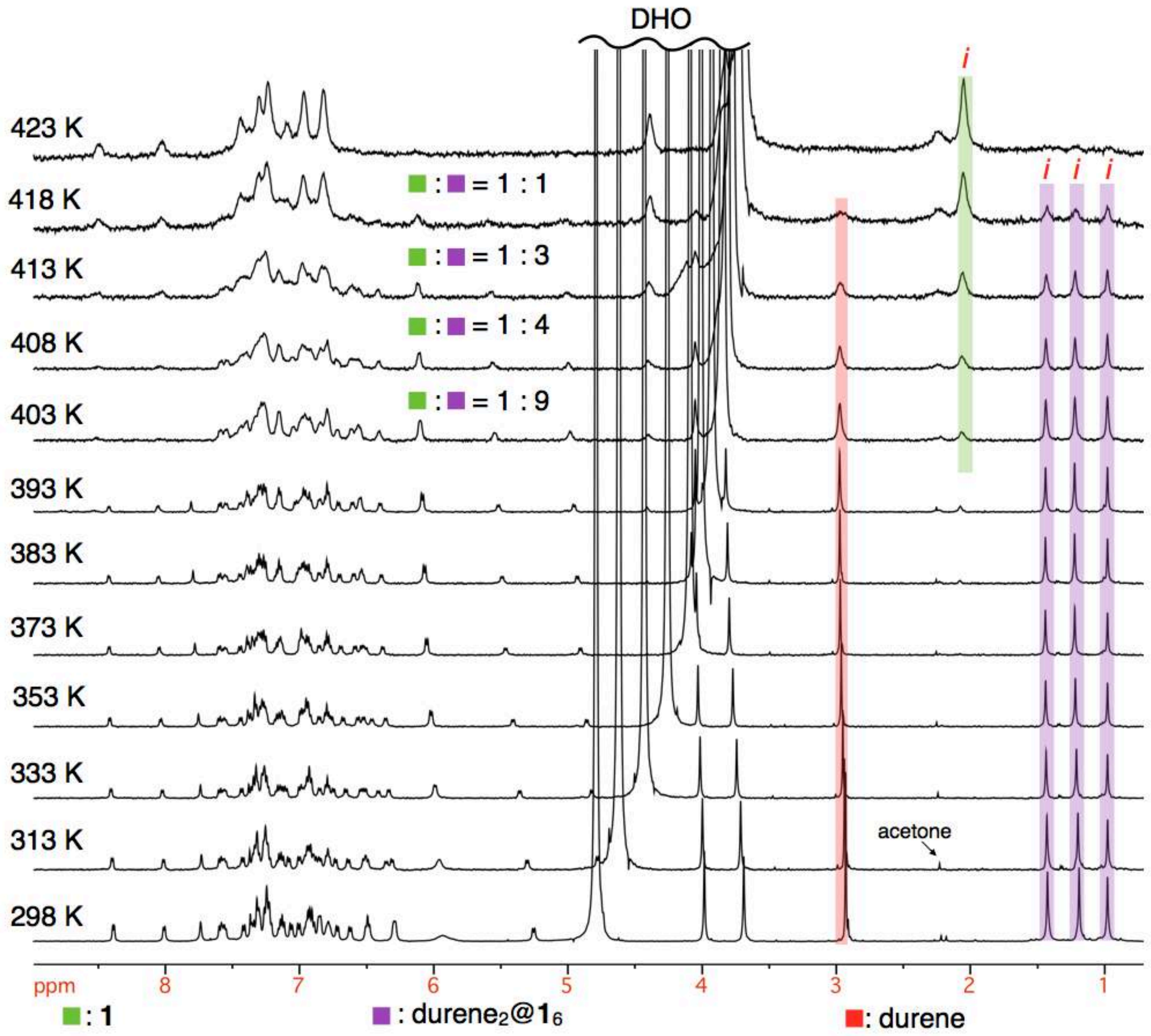

Supplementary Figure 19. Variable temperature ^{1}H NMR spectra of DU$_2$@**1**$_6$ (500 MHz, D$_2$O, [**1**]$_{total}$ = 1.0 mM). DU indicates durene.

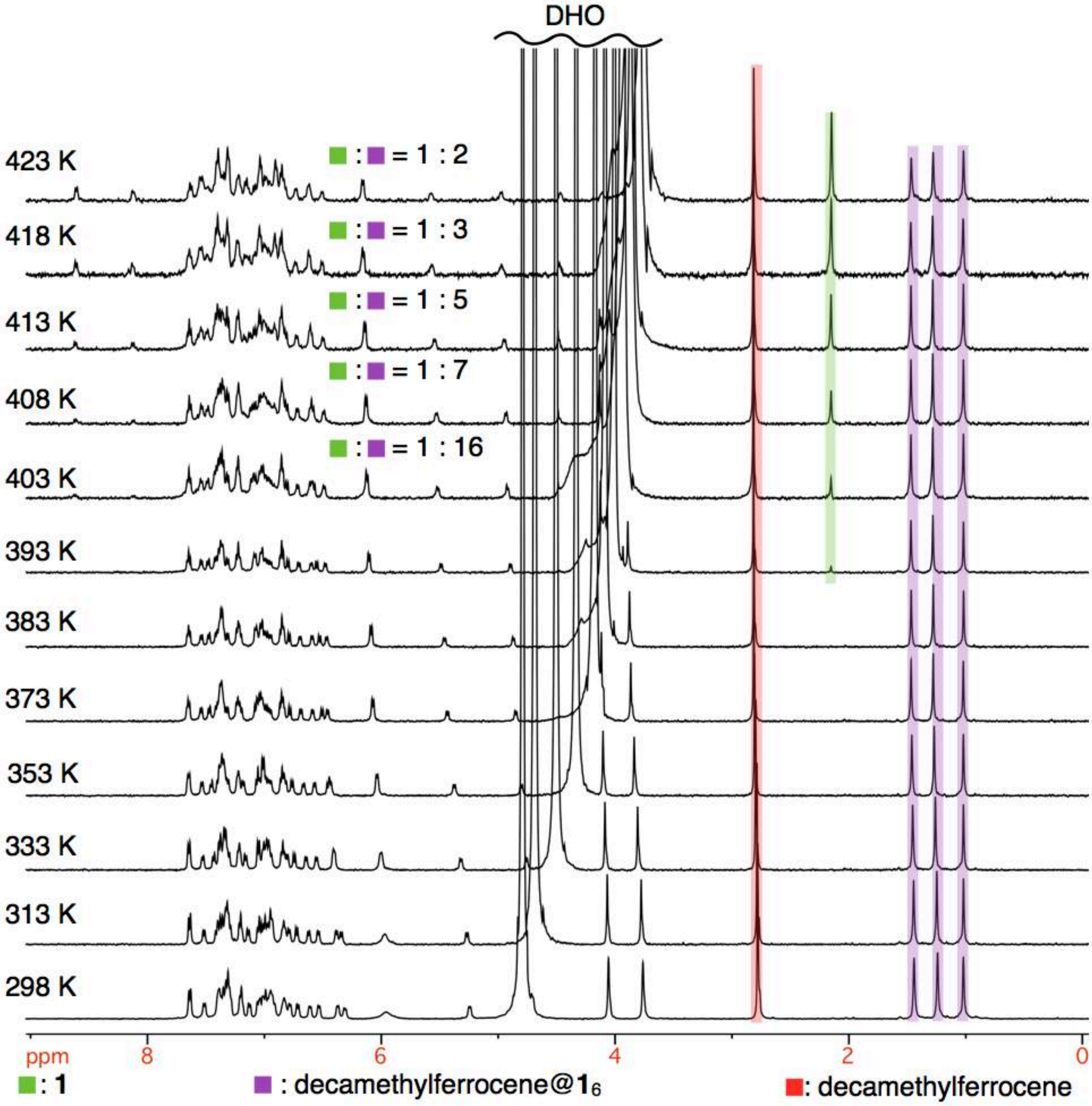

Supplementary Figure 20. Variable temperature ^{1}H NMR spectra of DF@$\mathbf{1}_6$ (500 MHz, D_2O, $[\mathbf{1}]_{total}$ = 1.0 mM). DF indicates decamethylferrocene.

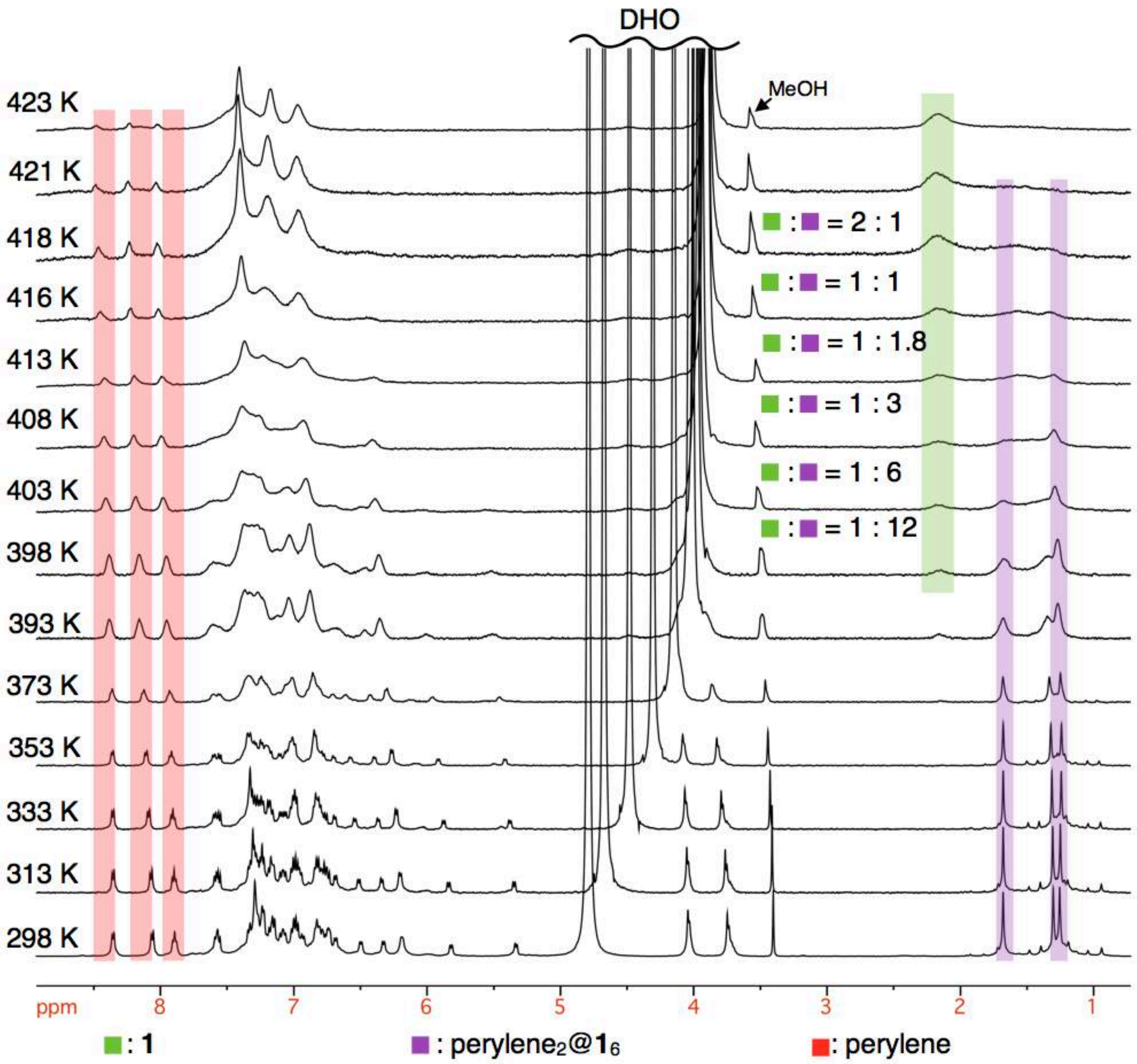

Supplementary Figure 21. Variable temperature ^{1}H NMR spectra of $PE_2@\mathbf{1}_6$ (500 MHz, D_2O, $[\mathbf{1}]_{total}$ = 1.0 mM). PE indicates perylene.

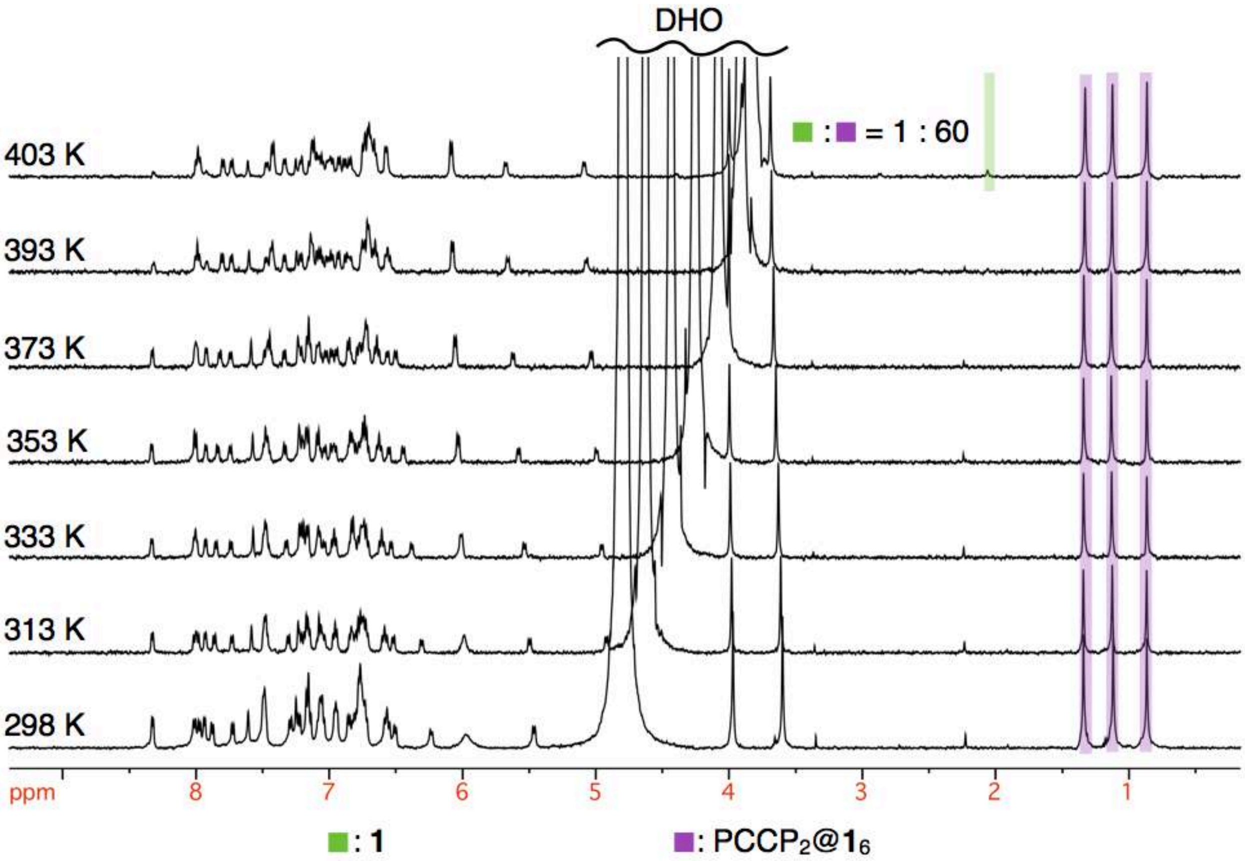

Supplementary Figure 22. Variable temperature ^{1}H NMR spectra of $PCCP_2@\mathbf{1}_6$ (500 MHz, D_2O, $[\mathbf{1}]_{total}$ = 1.0 mM). PCCP indicates pentacyanocyclopentadienide

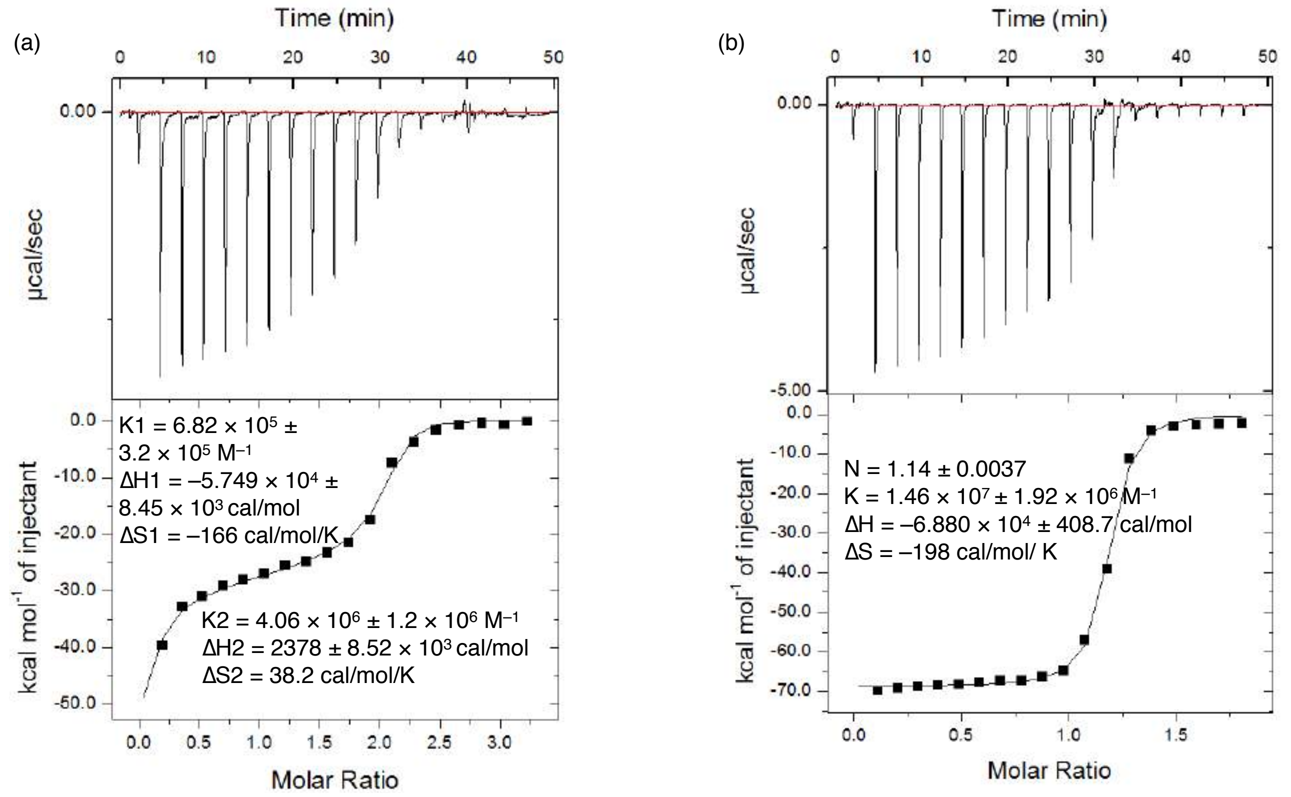

Supplementary Figure 23. ITC titration curves for the encapsulation of anionic guests (PCCP or CB) in the nanocube obtained at 298 K. (a) A titration of PCCP (0.5 mM) to $\mathbf{1}_6$ ($[\mathbf{1}]_{\text{total}}$ = 0.2 mM). Integrations of the heat released were fitted to a sequential binding model with two binding sites. As a result of fitting analysis, the thermodynamic parameters were determined as follows: ΔG_1 = –8.0 kcal mol^{-1}, ΔH_1 = –57.5 kcal mol^{-1}, $T\Delta S_1$ = –49.5 kcal mol^{-1}, ΔG_2 = –9.0 kcal mol^{-1}, ΔH_2 = 2.4 kcal mol^{-1}, $T\Delta S_2$ = 11.4 kcal mol^{-1}. (b) A titration of CB (0.3 mM) to $\mathbf{1}_6$ ($[\mathbf{1}]_{\text{total}}$ = 0.2 mM). Integrations of the heat released were fitted to a 1:1 binding model. As a result of fitting analysis, the thermodynamic parameters were determined as follows: ΔG = –9.8 kcal mol^{-1}, ΔH = –68.8 kcal mol^{-1}, $T\Delta S$ = –59.0 kcal mol^{-1}.

Supplementary Tables

Supplementary Table 1. Encapsulation behavior of $\mathbf{1}_6$

guest	volume (Å^3)	length of longest distance (Å)	Number of guests	$\Delta i^{1\,a}$ (ppm)	$\Delta i^{2\,a}$ (ppm)	$\Delta i^{3\,a}$ (ppm)	total Shift $\Delta\delta^b$ (ppm)	volume × encapsulate number (Å^3)
**toluene**	112	8.3	3	0.117	0.079	0.149	0.345	336
***o*-xylene**	134	8.3	2	0.059	0.055	0.085	0.199	268
***p*-xylene**	133	9.3	2	0.074	0.064	0.085	0.223	266
***m*-xylene**	135	8.8	3	0.143	0.079	0.187	0.409	405
**naphthalene**	139	9.6	2	0.073	0.056	0.070	0.199	278
**mesitylene**	158	8.8	2	0.081	0.064	0.049	0.194	316
**durene (DU)**	174	9.3	2	0.102	0.071	0.107	0.280	348
**anthracene**	187	12.0	2	0.224	0.062	0.165	0.450	374
**1,3,5-tribromo mesitylene (TBM)**	220	9.8	2	0.113	0.081	0.199	0.393	440
**hexamethyl benzene**	216	9.3	2	0.131	0.079	0.182	0.392	432
**1,3,5-triiodo mesitylene**	246	10.2	2	0.177	0.181	0.224	0.582	492
**[2.2]paracyclo-phane (PC)**	248	9.8	1	0.056	0.049	0.065	0.170	248
**perylene (PE)**	254	12.0	2	0.301	0.129	0.331	0.760	508
**decamethyl ferrocene (DF)**	407	10.8	1	0.097	0.099	0.129	0.325	407
***n*-propane**	74	6.8	3	0.097	0.069	0.124	0.290	222
***n*-butane**	95	8.0	3	0.090	0.071	0.114	0.275	285
***n*-pentane**	114	8.6	3	0.109	0.069	0.133	0.311	343
***n*-hexane**	137	9.4	3	0.152	0.094	0.180	0.426	411
**cyclohexane**	122	7.4	3	0.141	0.059	0.177	0.377	367
***n*-heptane**	157	11.1	2	0.084	0.058	0.114	0.256	314
***n*-octane**	179	13.2	2	0.098	0.064	0.129	0.291	358
***n*-decane**	223	15.7	2	0.159	0.092	0.200	0.451	445
***n*-dodecane**	265	18.3	1	0.060	0.045	0.084	0.189	265
***n*-tetradecane**	307	20.8	1	0.087	0.059	0.109	0.255	307
***n*-hexadecane**	351	23.4	1	0.103	0.063	0.129	0.295	351
***n*-octadecane**	394	25.9	1	0.127	0.077	0.161	0.365	394
***n*-eicosane**	438	28.5	1	0.156	0.087	0.205	0.448	438
***n*-docosane**	482	31.1	1	0.193	0.106	0.256	0.555	482
***n*-tetracosane**	535	33.6	1	0.236	0.125	0.300	0.661	535
**PCCP**	193	10.3	2	–0.002	–0.019	–0.023	–0.044	386
**CB**	403	9.1	1	–0.038	0.007	0.009	–0.022	403
**methane**	30	4.2	0					
**ethene**	42	5.5	0					
**naphthacene**	235	14.3	0					
***p*-di-*tert*-butylbenzene**	287	12.0	0					
**coronene**	325	12.6	0					
**fullerene**	533	10.4	0					
**tetra-*n*-butyl ammonium bromide**	446	13.5	0					

[a] $\Delta i^1 = \delta(i^1)$ of $G_n@\mathbf{1}_6$ (G: guest molecule) – $\delta(i^1)$ of $\mathbf{1}_6$. $\Delta i^2 = \delta(i^2)$ of $G_n@\mathbf{1}_6$ (G: guest molecule) – $\delta(i^2)$ of $\mathbf{1}_6$. $\Delta i^3 = \delta(i^3)$ of $G_n@\mathbf{1}_6$ (G: guest molecule) – $\delta(i^3)$ of $\mathbf{1}_6$.

[b] Total chemical shift change of the *p*-tolyl methyl signals. $\Delta\delta = \Delta i^1 + \Delta i^2 + \Delta i^3$

Supplementary Table 2. Free energy changes for the formation of host-guest complexes of $\mathbf{1}_6$, ΔG_a, around $T_{1/2}$ in D_2O.

Nanocube complexes	GSA / mM	T / K	Formation ratio ([monomer] / 6[hexamer])	K_a / M^{-5}	ΔG_a / kJ mol^{-1}	$-\Delta G$ / kJ mol^{-1}
PC@$\mathbf{1}_6$	1.0	403	1/6	1.7×10^{19}	–148	148
		408	1/5	2.1×10^{18}	–143	143
		413	1	5.3×10^{15}	–124	124
		415	1.2	2.9×10^{15}	–123	123
		423	5	8.3×10^{13}	–113	113
DU_2@$\mathbf{1}_6$	1..0	403	1/9	1.5×10^{20}	–156	156
		408	1/4	2.1×10^{18}	–143	143
		413	1/2	8.1×10^{16}	–134	134
		418	1	5.3×10^{15}	–126	126
DF@$\mathbf{1}_6$	1.0	403	1/16	3.8×10^{21}	–166	166
		408	1/7	3.8×10^{19}	–153	153
		413	1/5	6.5×10^{18}	–149	149
		418	1/3	5.1×10^{17}	–142	142
		423	1/2	8.1×10^{16}	–137	137
PE_2@$\mathbf{1}_6$	1.0	398	1/12	7.4×10^{20}	–159	159
		403	1/6	1.7×10^{19}	–148	148
		408	1/3	5.1×10^{17}	–138	138
		413	1/1.8	5.2×10^{16}	–132	132
		416	1	5.3×10^{15}	–125	125
		418	2	6.3×10^{14}	–118	118
$PCCP_2$@$\mathbf{1}_6$	1.0	403	1/60	8.5×10^{24}	–192	192

*The temperature colored in red indicates the $T_{1/2}$ for the nanocube.

*$T_{1/2}$ value of DF@$\mathbf{1}_6$ is higher than 423 K, which is the highest limit of our NMR instrument.

Supplementary Table 3. longitudinal relaxation time (T_1) of the **1** and $\mathbf{1}_6$ nanocubes

Compounds	$T_1{}^a$ / s
$\mathbf{1}^b$	0.908
$\mathbf{1}_6$	2.148
$PCCP_2$@$\mathbf{1}_6$	2.560

$^a T_1$ is the average of T_1 values of the *p*-tolyl methyl groups, i^1, i^2, and i^3.

$^b T_1$ value of the **1** is measured in CD_3OD.
